# Novel Cholinesterase Inhibitors Based on *O*-Aromatic *N*,*N*-Disubstituted Carbamates and Thiocarbamates

**DOI:** 10.3390/molecules21020191

**Published:** 2016-02-11

**Authors:** Martin Krátký, Šárka Štěpánková, Katarína Vorčáková, Markéta Švarcová, Jarmila Vinšová

**Affiliations:** 1 Department of Inorganic and Organic Chemistry, Faculty of Pharmacy, Charles University, Heyrovského 1203, 500 05 Hradec Králové, Czech Republic; Marketa.Svarcova@ujep.cz (M.Š.); vinsova@faf.cuni.cz (J.V.); 2 Department of Biological and Biochemical Sciences, Faculty of Chemical Technology, University of Pardubice, Studentská 573, 532 10 Pardubice, Czech Republic; Sarka.Stepankova@upce.cz (Š.Š.); Katarina.Vorcakova@upce.cz (K.V.); 3 Faculty of Science, J. E. Purkinje University, České mládeže 8, 400 96 Ústí nad Labem, Czech Republic

**Keywords:** acetylcholinesterase, butyrylcholinesterase, carbamate, enzyme inhibition, salicylanilide, thiocarbamate

## Abstract

Based on the presence of carbamoyl moiety, twenty salicylanilide *N*,*N*-disubstituted (thio)carbamates were investigated using Ellman’s method for their ability to inhibit acetylcholinesterase (AChE) and butyrylcholinesterase (BChE). *O*-Aromatic (thio)carbamates exhibited weak to moderate inhibition of both cholinesterases with IC_50_ values within the range of 1.60 to 311.0 µM. IC_50_ values for BChE were mostly lower than those obtained for AChE; four derivatives showed distinct selectivity for BChE. All of the (thio)carbamates produced a stronger inhibition of AChE than rivastigmine, and five of them inhibited BChE more effectively than both established drugs rivastigmine and galantamine. In general, 5-chloro-2-hydroxy-*N*-[4-(trifluoromethyl)-phenyl]benzamide, 2-hydroxy-*N*-phenylbenzamide as well as *N*-methyl-*N*-phenyl carbamate derivatives led to the more potent inhibition. *O*-{4-Chloro-2-[(4-chlorophenyl)carbamoyl]phenyl} dimethylcarbamothioate was identified as the most effective AChE inhibitor (IC_50_ = 38.98 µM), while 2-(phenylcarbamoyl)phenyl diphenylcarbamate produced the lowest IC_50_ value for BChE (1.60 µM). Results from molecular docking studies suggest that carbamate compounds, especially *N*,*N*-diphenyl substituted representatives with considerable portion of aromatic moieties may work as non-covalent inhibitors displaying many interactions at peripheral anionic sites of both enzymes. Mild cytotoxicity for HepG2 cells and consequent satisfactory calculated selectivity indexes qualify several derivatives for further optimization.

## 1. Introduction

Acetylcholine (ACh) is a cholinergic neurotransmitter interacting with either nicotinic or muscarinic receptors, e.g., at all autonomic ganglia, neuromuscular junction, the preganglionic sympathetic and parasympathetic neurons, in all the parasympathetic innervated organs, adrenal medulla, and at the sweat glands. The effect of ACh in synapses is terminated by the action of acetylcholinesterase (AChE; EC 3.1.1.7) and butyrylcholinesterase (pseudocholinesterase or plasma cholinesterase; BChE; EC 3.1.1.8), which hydrolyse ACh rapidly into choline and acetate. However, AChE remains the major cholinesterase within the human brain. This enzyme exists in multiple molecular forms with similar catalytic properties. The esteratic site, where ACh is hydrolysed, contains the catalytic triad of three amino acids: serine, histidine and glutamate [1,2,3].

Degeneration of the cholinergic projection from the *nucleus basalis Meynerti* to the forebrain neocortex and associated limbic structures is one of the pathologies associated with Alzheimer’s disease (AD) [3], the most common type of dementia. Unfortunately, the number of patients suffering from this disease has increased continuously during the last decades. Based on cholinergic hypothesis, inhibition of cholinesterases (ChE) represents the mainstay of pharmacotherapy of AD. During the progression of AD, BChE activity and BChE/AChE ratio are increased thus compensating the loss of AChE activity. BChE can substitute for AChE constitutively. That is why BChE inhibition may provide a desirable feature of AD therapy [1,4]. Current drugs for the treatment of AD are non-selective (rivastigmine) or AChE-selective inhibitors (galantamine, donepezil) [1,5]. However, these three ChE inhibitors possess clinically comparable efficacy and side-effect profiles. It is unclear whether additional inhibition of BCHE by rivastigmine or galantamine's allosteric modulation of nicotinic receptors offer any extra clinical benefit [6]. In addition to increased ACh levels and symptomatic benefit, recent findings indicate that ChE inhibitors can attenuate neuronal damage and protect them from cellular death. This way might affect AD pathogenesis and slow down the progression [7], but there is still no really effective causative therapy.

**Figure 1:**
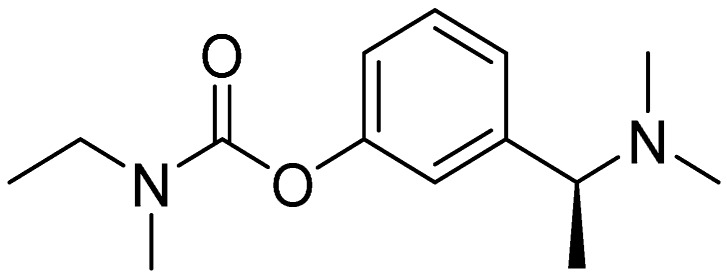
Rivastigmine.

It has been demonstrated that carbamate moiety is a pharmacophore for ChE inhibition activity. Various carbamates have been proposed and proven as highly efficient AChE and BChE inhibitors [8,9,10,11,12]. Also rivastigmine ((*S*)-3-[1-(dimethylamino)ethyl]phenyl ethyl(methyl)carbamate; Figure 1 used in the treatment of AD and some other dementias belong to aromatic carbamates. In addition to this use, carbamates have been used in human medicine (e.g., for the treatment of myasthenia gravis, glaucoma), veterinary medicine, agriculture, and for military purposes [3].

The mechanism of action of carbamates is distinct from the majority of organophosphorus-based inhibitors, although both these groups are substrate analogues of ACh. They enter the active site and affect the esteratic site of ChE by covalent interaction (phosphorylation or carbamoylation) with serine hydroxyl providing more stable esters than those resulted from physiological reaction (acetylation) with ACh. The principal difference between organophosphates and carbamates consists in the stability of the ChE-inhibitor complex. Organophosphates phosphorylate serine residues in a non-reversible way and the regeneration of the enzyme is very slow (irreversible inhibition). Less stable carbamoyl-serine complex can be split by spontaneous hydrolysis markedly faster (pseudo-irreversible inhibition) [3,13]. Depending on the structure of carbamates, ChE inhibition can be also irreversible or non-covalent [10,14,15].

Salicylanilide derivatives exhibit a wide range of interesting biological properties including inhibition of various enzymes [16,17,18,19,20,21,22]. Previously, we identified salicylanilide *O*,*O*-diethyl thiophosphates (phosphorothioates) [23] and their oxo analogues (diethyl phosphates) [24] as pseudo-irreversible inhibitors of both AChE and BChE with low micromolar IC_50_ values. Interestingly, thiophosphates exhibited the balanced activity against both cholinesterases [23] whereas phosphates showed more effective inhibition of BChE [24]. Imramovský *et al.* [14] reported inhibition of AChE by salicylanilide *N*-alkyl carbamates and their molecular docking.

**Figure 2:**
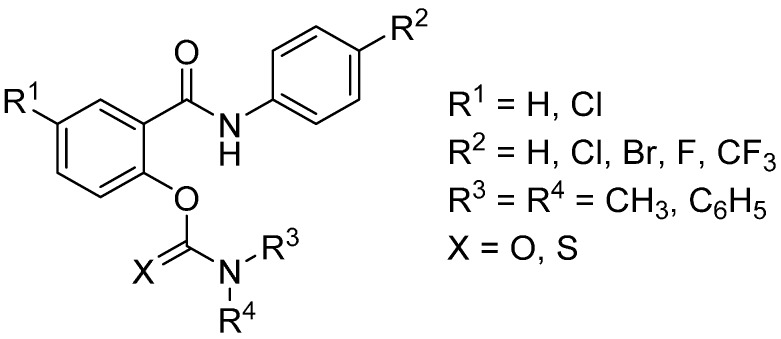
Salicylanilide (thio)carbamates **1a**–**5d**.

Keeping in mind these facts about carbamates and salicylanilides, we evaluated salicylanilide *N*,*N*-disubstituted (thio)carbamates as potential inhibitors of both ChE. Previously, these (2-(phenylcarbamoyl)phenyl disubstituted(thio)carbamate molecules (their general structure is depicted in Figure 2) exhibit *in vitro* antimicrobial activity against mycobacteria, Gram-positive cocci and *Trichophyton mentagrophytes*. In contrast to parent salicylanilides, these compounds offer a lower cytotoxicity and a higher lipophilicity expressed as calculated log*P* values [19]. Increased log*P* may help to improve poor bioavailability of salicylanilides and to facilitate permeability through the biological membranes [16]. More lipophilic derivatives are also able to enter the central nervous system via passive diffusion more easily and/or readily.

## 2. Results and Discussion

### 2.1. In Vitro Inhibition of Acetylcholinesterase and Butyrylcholinesterase

The IC_50_ values were determined using the spectrophotometric Ellman’s method, which is a simple and rapid method to determine the SH and -S-S- groups content in proteins [25]. This method is widely used for the evaluation of cholinesterase activity and screening the efficiency of ChE inhibitors. Cholinesterase activity is measured indirectly by quantifying the concentration of the 5-mercapto-2-nitrobenzoic acid ion formed in the reaction between the thiol reagent 5,5′-dithiobis(2-nitrobenzoic acid) and thiocholine, a product of substrate hydrolysis (*i.e.*, acetylthiocholine, ATCh) by cholinesterases [26]. Ellman’s method was modified slightly according to Zdrazilova *et al.* [27].

The inhibitory potency of fifteen carbamates and five thiocarbamates was evaluated for AChE from electric eel and BChE from equine serum. The efficacy of the screened compounds is expressed as IC_50_ values, representing the concentration of an inhibitor required for 50% inhibition of the enzyme. The results were compared with rivastigmine and galantamine, two established anti-dementia drugs with different structures and mechanisms of action. Rivastigmine is an acylating pseudo-irreversible carbamate-based inhibitor affecting the function of both cholinesterases, while galantamine acts as a non-acylating competitive reversible inhibitor. Furthermore, it modulates allosterically nicotinic ACh receptors.

The synthesis of (thio)carbamates **1a**–**5d** was reported previously by Krátký *et al.* [19].

All of the (thio)carbamates **1a**–**5d** showed weak to moderate inhibition of both cholinesterases with IC_50_ values from 1.60 to 311.0 µM (Table 1). The IC_50_ values of all tested derivatives for inhibition of AChE were in a comparatively narrow concentration range of 38–90 µM. *O*-{4-Chloro-2-[(4-chlorophenyl)carbamoyl]phenyl} dimethylcarbamothioate (**1b**) was identified as the most effective AChE inhibitor (IC_50_ = 38.98 µM), whereas *O*-{4-chloro-2-[(4-fluorophenyl)carbamoyl]phenyl} dimethylcarbamothioate (**3b**) produced the least inhibition with IC_50_ of 89.74 µM. In general, it is possible to conclude that better effectiveness was achieved by the 4-(trifluoromethyl)aniline and 5-chlorosalicylic acid derivatives **4a**–**d**, although compared to other derivatives the IC_50_ value differences are not enormous. That is why there is no clear influence of carbamate nitrogen substitution pattern on the activity. The removal of 5-chlorine atom from salicylic moiety is well tolerated without any increase of IC_50_ values (derivatives **5** *vs.* **1**–**4**) similarly to the replacement of carbamoyl by thiocarbamoyl group and *vice versa* (compounds **b** *vs.* **a**, **c**, **d**). Nevertheless, regarding a large number of agents with their activity in low micromolar, submicromolar and nanomolar concentrations [2,8,12], investigated derivatives **1a**–**5d** could be considered to be only weak AChE inhibitors.

**Table 1:** IC_50_ values for acetylcholinesterase and butyrylcholinesterase. 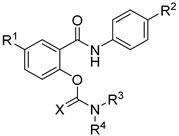

Code	R^1^	R^2^	R^3^	R^4^	X	IC_50_ for AChE [µM]	IC_50_ for BChE [µM]	Selectivity to BChE
**1a**	Cl	Cl	CH_3_	CH_3_	O	72.87 ± 1.06	88.42 ± 1.27	0.8
**1b**	Cl	Cl	CH_3_	CH_3_	S	**38.98 ± 1.11**	51.38 ± 1.65	0.8
**1c**	Cl	Cl	CH_3_	Ph	O	74.83 ± 2.95	**4.20 ± 0.18**	**17.8**
**1d**	Cl	Cl	Ph	Ph	O	54.81 ± 1.43	106.46 ± 3.64	0.5
**2a**	Cl	Br	CH_3_	CH_3_	O	62.38 ± 1.36	54.21 ± 0.30	1.2
**2b**	Cl	Br	CH_3_	CH_3_	S	59.05 ± 2.13	54.64 ± 0.42	1.1
**2c**	Cl	Br	CH_3_	Ph	O	81.60 ± 2.92	**4.30 ± 0.51**	**19.0**
**2d**	Cl	Br	Ph	Ph	O	63.29 ± 0.95	144.80 ± 29.02	0.4
**3a**	Cl	F	CH_3_	CH_3_	O	76.17 ± 2.87	311.00 ± 4.50	0.2
**3b**	Cl	F	CH_3_	CH_3_	S	89.74 ± 4.68	91.37 ± 5.45	1.0
**3c**	Cl	F	CH_3_	Ph	O	62.54 ± 5.03	**7.34 ± 0.14**	8.5
**3d**	Cl	F	Ph	Ph	O	**49.03 ± 3.88**	15.63 ± 0.11	3.1
**4a**	Cl	CF_3_	CH_3_	CH_3_	O	**49.04 ± 0.54**	28.14 ± 0.86	1.7
**4b**	Cl	CF_3_	CH_3_	CH_3_	S	**41.98 ± 0.55**	36.22 ± 0.28	1.2
**4c**	Cl	CF_3_	CH_3_	Ph	O	**49.85 ± 2.76**	**1.97 ± 0.02**	**25.3**
**4d**	Cl	CF_3_	Ph	Ph	O	**47.39 ± 2.78**	49.00 ± 0.17	1.0
**5a**	H	H	CH_3_	CH_3_	O	57.74 ± 1.60	83.59 ± 6.84	0.7
**5b**	H	H	CH_3_	CH_3_	S	61.94 ± 6.56	26.62 ± 2.78	2.3
**5c**	H	H	CH_3_	Ph	O	55.01 ± 2.46	11.38 ± 0.48	4.8
**5d**	H	H	Ph	Ph	O	53.79 ± 4.26	**1.60 ± 0.01**	**33.6**
**Rivastigmine**						501 ± 3.08	19.95 ± 0.20	25.1
**Galantamine**						4 ± 0.13	7.96 ± 0.59	0.5

AChE and BChE inhibition is expressed as the mean ± SD (*n* = 3 experiments). Selectivity to BChE: IC50 for AChE/IC50 for BChE. The best results for each enzyme and favourable selectivity are shown in bold.

In contrast to AChE, IC_50_ values for BChE are in a broader range of 1.60 to 311.0 µM. Among all of the tested compounds, 2-(phenylcarbamoyl)phenyl diphenylcarbamate (**5d**) was found to be the most potent BChE inhibitor *in vitro* (IC_50_ value of 1.60 µM) and the least effective inhibitor was 4-chloro-2-[(4-fluorophenyl)carbamoyl]phenyl dimethylcarbamate (**3a**, IC_50_ = 311.0 µM). The most active BChE inhibitors displayed moderate inhibitory action, remaining activities are rather weak. Similarly to AChE inhibition, improved activity towards BChE was shown by derivatives of 4-(trifluoromethyl)aniline **4a**–**d**; their IC_50_ values did not exceed the value of 50 µM. Moreover, this group includes the second best inhibitor of BChE from all of the tested molecules, methyl(phenyl)carbamate **4c** with IC_50_ of 1.97 µM. Aniline ring substitution by 4-chlorine or 4-bromine produced a similar activity, which was superior to 4-fluoroaniline derivatives. Interestingly, non-halogenated (thio)carbamates **5** exhibited also a significant inhibition suggesting that halogenation of both salicylic and aniline rings is not necessary for the activity. Focusing on the various (thio)carbamate types used for the modification of halogenated salicylanilides, the compounds were ordered based on decreasing inhibition activity against BChE as follows: *N*-methyl-*N*-phenyl carbamates **c** >˃ *N*,*N*-dimethyl thiocarbamates **b** > *N*,*N*-diphenyl carbamates **d** and *N*,*N*-dimethyl carbamates **a**. For the majority of derivatives, the change of *N*,*N*-dimethyl carbamates **a** to thiocarbamates **b** as well as the replacement of one *N*-methyl by phenyl increased the activity.

When concentrated on selectivity to ChE (Table 1), (thio)carbamates can be classified into three groups. Members of first group are more effective inhibitors of AChE (selectivity to BChE of ≤0.5; **1d**, **2d**, **3a**). Second, the largest group embraces compounds with balanced inhibition of both enzymes (selectivity within the range of 0.5 to 2.0): **1a**, **1b**, **2a**, **2b**, **3b**, **4a**, **4b**, **4d**, **5a**, in general *N*,*N*-dimethyl (thio)carbamates. Remaining derivatives including all *N*-methyl-*N*-phenyl carbamates are stronger inhibitors of BChE: **1c**, **2c**, **3c**, **3d**, **4c**, **5b**-**d**. Selectivity of four of them (**1c**, **2c**, **4c** and especially **5d**) towards BChE exceeded the value of 10, thus being comparable with rivastigmine.

The activities of salicylanilide derivatives **1a**–**5d** were compared with those of galantamine and rivastigmine. All of the tested derivatives showed a greater inhibition of AChE than rivastigmine but not galantamine, and five of them (**1c**, **2c**, **3c**, **4c** and **5d**) inhibited BChE more effectively than both drugs; additionally, two carbamates (**3d**, **5c**) exhibited superiority to rivastigmine only.

Drawing a comparison between *N*,*N*-disubstituted (thio)carbamates **1a**–**5d** and previously reported salicylanilide-based cholinesterases inhibitors, *N*-alkyl carbamates [14] demonstrated equal or almost equal IC_50_ values for AChE. Salicylanilide diethyl phosphates [24] showed a similar activity against AChE, but they inhibited BChE slightly stronger and more selectively. Diethyl thiophosphates (phosphorothioates) were superior towards AChE and they exhibited a comparatively closer concentration range for the inhibition of BChE. However, the most active carbamates **1c**, **2c**, **3c**, **4c**, **5d** showed lower IC_50_ values than the most active thiophosphates.

### 2.2. Cytotoxicity and Selectivity Indexes of (Thio)Carbamates

We also investigated selectivity indexes (SI) for (thio)carbamates inhibitors. SI is calculated as a ratio of cellular toxicity and IC_50_ values (in µM) for enzymes, and SI value higher than 10 indicates a rather acceptable toxicity (based on the analogy of the therapeutic index). Cytotoxicity was determined in human hepatocellular carcinoma (HepG2) cell model previously [19]. For the calculation of selectivity indexes, we selected several carbamates and thiocarbamates. First, the most effective inhibitors of AChE and BChE were involved (**1b**, **4b** and **1c**, **2c**, **3c**, **4c**, **5d**, respectively) and then two carbamates (**4a**, **5a**) with the lowest toxicity were added (Table 2).

In general, all SI values for thiocarbamates **1b** and **4b** are poor due to their comparatively higher cytotoxicity (83.51 and 67.29 µM, respectively). Selectivity indexes of **1c**, **3c**, **4b**, **4c** and **5d** for this enzyme did not reach breakpoint of 10 due to the lower activity against AChE. On the other hand, two dimethylcarbamates with IC_50_ values ~6 mM (**4a**, **5a**) provided a satisfactory selectivity (≥103.7), followed by *N*-methyl-*N*-phenyl carbamate **2c** (SI of 11.6). With respect to BChE, seven derivatives from total nine showed sufficient SI values within the range of ˃50.8 with superiority of 2-[(4-bromophenyl)carbamoyl]-4-chlorophenyl methyl(phenyl)carbamate (**2c**) and 4-chloro-2-{[4-(trifluoro-methyl)phenyl]carbamoyl}phenyl dimethylcarbamate (**4a**, SI ≥ 219.8), thus indicated a safety of investigated derivatives.

**Table 2:** Cytotoxicity and selectivity indexes for selected (thio)carbamates.

Code	IC_50_ for HepG2 [µM] [19]	Selectivity Index for AChE	Selectivity Index for BChE
**1b**	83.51	2.1	1.6
**1c**	289.0	3.9	**68.8**
**2c**	945.1	**11.6**	**219.8**
**3c**	512.0	8.2	**69.8**
**4a**	6508.0	**132.7**	**231.3**
**4b**	67.29	1.6	1.9
**4c**	˃100	˃2.0	**˃50.8**
**5a**	5985.9	**103.7**	**71.6**
**5d**	211.1	3.9	**131.9**

Selectivity indexes higher than 10 are shown in bold.

### 2.3. Molecular Docking

In order to elucidate possible mode of binding, molecular docking was performed using crystallographic structures of human AChE (pdb code 4PQE) and human BChE (pdb code 1POI). In the preparation process all water molecules were removed from the enzymes and structures of enzymes and ligands were optimized using UCSF Chimera software package (Amber force field) [28]. Docking was performed using Autodock Vina [29] and Autodock 4.2 [30]. The molecules of enzymes were partly flexible. The 3D affinity grid box was designed to include the full active and peripheral site of AChE and BChE.

Generally, carbamate compounds are considered to be covalent inhibitors reacting directly with the active-site serine hydroxyl [3,13]. However, being rather spacious molecules and with their carbamate bond relatively inaccessible for the direct interaction with the enzyme (especially for diphenyl substituted carbamates **d**), compounds **1a**–**5d** prepared in our laboratory could also act as non-covalent or allosteric inhibitors. Moreover, the presence of four aromatic moieties could be beneficial for the formation of interactions in the active gorge of AChE which is lined predominantly by aromatic amino acid residues or interactions with the peripheral anionic site (PAS) of both AChE and BChE. Additionally, some carbamates have been also reported as the PAS-directed irreversible inhibitors of AChE [15] or proposed as competitive acting agents [14].

The structures of AChE and BChE active sites have been studied extensively and are described in detail [31,32,33]. Active sites are buried under the surface of the molecules at the bottom of a relatively narrow gorge [31]. Though both enzymes share considerable structure similarities, there are significant differences resulting in distinct susceptibility to substrates and modulators of activity.

The active gorge of AChE is lined with aromatic acid residues serving for the navigation of positively charged molecules towards the esteratic site. In close proximity of catalytic triad Ser-His-Glu, several aromatic acid residues (e.g., Trp86, Phe337) can be found, forming so-called α-anionic site [32]. Moreover, on the outer rim of the cavity, another region consisting of predominantly aromatic residues (Trp286, Tyr72, Tyr124) comprises peripheral anionic site. This structure feature is important for the function of allosteric inhibitors [34]. The active site of BChE has very similar organisation, though its cavity is composed mostly of hydrophobic residues of aliphatic amino acids which leaves more space in the gorge increasing thus the ability to accommodate larger molecules with bulkier substituents [35,36].

Molecular modelling studies performed on AChE with the series of salicylanilide *N*,*N*-disubstituted (thio)carbamates **1a**–**5d** suggested that these compounds fill the cavity of the enzyme with aniline part of the molecule heading out of the gorge, salicylic aromatic ring in close proximity to Trp86 of α-anionic site and amide group pointing towards Ser203 hydroxyl. All of the molecules displayed a similar orientation though diphenyl substituted carbamates (**1a**–**5d**) stayed closer to the enzyme surface showing additional interactions with PAS.

**Figure 3:**
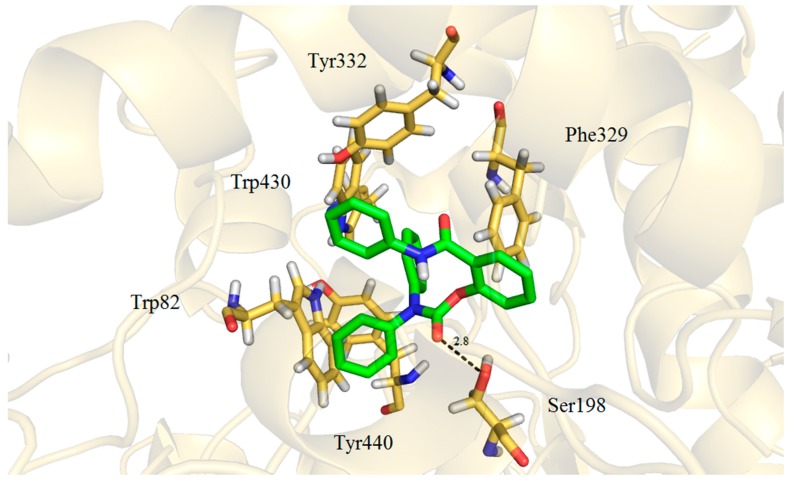
Compound **5d** in active site of BChE together with interacting residues.

Two modes of binding were observed in the active site of BChE. *N*,*N*-Dimethyl carbamates **a**, thiocarbamates **b** and one representative of *N*-methyl-*N*-phenyl carbamate (**5c**) showed a similar orientation spreading across the cavity, with salicylic part of the molecule showing π-π interactions with Trp82 and Tyr332 and aniline ring displaying π-π interactions with Phe329 (T-stacking) and amide bond in relatively close proximity to Ser198 (O-N = 3.20 Å for the compound **5c**). *N*,*N*-Diphenyl carbamates **d** and some of *N*-methyl-*N*-phenyl carbamates **c** appeared to be moved slightly out of the cavity blocking substantially the entrance to the gorge. Diphenylcarbamoyl part of the molecule fills up a small aromatic hollow formed by Trp82, Tyr332, Trp430 and Tyr440, carbonyl oxygene is in close proximity to Ser198 (O-O = 2.83 Å), salicylic moiety shows a possible interaction with Phe329 and aniline part of the molecule is heading out of the gorge (Figure 3).

## 3. Experimental Section

### 3.1. Determination of Cholinesterase Inhibition

The IC_50_ values were determined using the spectrophotometric Ellman’s method [25,26]. All of the tested compounds were dissolved in 0.01 M dimethyl sulphoxide and then diluted in demineralised water to 0.001 M and 0.0001 M. Ellman’s method was modified slightly according to Zdrazilova *et al.* [27]. Acetylcholinesterase was obtained from electric eel (*Electrophorus electricus* L.) and butyrylcholinesterase was from equine serum. Galantamine and rivastigmine were involved as reference drugs.

### 3.2. Molecular Docking

Molecular docking was performed using crystallographic structures of human AChE (pdb code 4PQE) and human BChE (pdb code 1POI). The 3D structures of ligands were prepared in Chem3D Pro 13.0 (a part of ChemBioOffice 2012 Package, CambridgeSoft, Cambridge, MA, USA). In the preparation process all water molecules were removed from the enzymes and structures of enzymes and ligands were optimized using UCSF Chimera software package (Amber force field) [28]. Docking was performed using Autodock Vina [29] and Autodock 4.2 [30] (a Lamarckian genetic algorithm was used). The molecules of enzymes were partly flexible. The 3D affinity grid box was designed to include the full active and peripheral site of AChE and BChE. The number of grid points in the *x*-, *y*- and *z*-axes was 24, 24 and 24 with grid points separated by 1 Å (Autodock Vina) and 40, 40 and 40 with grid points separated by 0.4 Å (Autodock 4).

## 4. Conclusions

Fifteen salicylanilide *N*,*N*-disubstituted carbamates and five *N*,*N*-dimethylthiocarbamates were evaluated for their inhibition of acetylcholinesterase from electric eel and butyrylcholinesterase from equine serum. All derivatives exhibited *in vitro* inhibition of both ChE with micromolar and low micromolar IC_50_ values. Several derivatives showed a promising activity especially against BChE. Eight derivatives were more selective against BChE, whereas remaining compounds produced mostly balanced inhibition of both enzymes. (Thio)carbamates share higher *in vitro* activity when compared to rivastigmine against AChE (IC_50_ values of ≤ 89.7 and 501 µM, respectively) and seven of them even against BChE (*i.e.*, with IC_50_ lower than 19.95 µM). *N*-Methyl-*N*-phenyl carbamates and derivatives of 5-chloro-2-hydroxy-*N*-[4-(trifluoromethyl)phenyl]benzamide were identified optimal for the enzyme inhibition. Selected salicylanilide *N*,*N*-disubstituted (thio)carbamates also exhibited satisfactory selectivity indexes for both cholinesterases as a consequence of their comparatively mild cytotoxicity.

Molecular docking studies performed on both enzymes suggest that these molecules may act as non-covalent inhibitors of AChE and BChE. Especially *N*,*N*-diphenyl substituted carbamates showed many possible interactions in the PAS blocking thus the entrance of the enzyme cavity. The preference of *N*-methyl-*N*-phenyl and *N*,*N*-diphenyl carbamates towards BChE at the expense of AChE springs from the differences in the active gorge arrangement. Though cavity of AChE contains a significant amount of aromatic acid residues, it is possible that BChE with more spacious cavity may be able to accommodate compounds with larger substituents optimally.

These results can stimulate a follow-up study as its starting point with the goal to reach more efficacious inhibitors with submicromolar activities keeping also the low cytotoxicity.

## Abbreviations

The following abbreviations are used in this manuscript:

ACh: acetylcholine
ATCh: acetylthiocholine
AD: Alzheimer’s disease
AChE: acetylcholinesterase
BChE: butyrylcholinesterase
ChE: cholinesterases
